# Availability of screening and treatment for common mental disorders in HIV clinic settings: data from the global International epidemiology Databases to Evaluate AIDS (IeDEA) Consortium, 2016–2017 and 2020

**DOI:** 10.1002/jia2.26147

**Published:** 2023-08-03

**Authors:** Angela M. Parcesepe, Melissa Stockton, Molly Remch, C. William Wester, Charlotte Bernard, Jeremy Ross, Andreas D. Haas, Rogers Ajeh, Keri N. Althoff, Leslie Enane, William Pape, Albert Minga, Edith Kwobah, Mpho Tlali, Junko Tanuma, Dominique Nsonde, Aimee Freeman, Stephany N. Duda, Denis Nash, Kathryn Lancaster, the IeDEA Consortium

**Affiliations:** ^1^ Department of Maternal and Child Health University of North Carolina at Chapel Hill Chapel Hill North Carolina USA; ^2^ University of North Carolina at Chapel Hill Carolina Population Center Chapel Hill North Carolina USA; ^3^ Department of Psychiatry Columbia University New York New York USA; ^4^ Department of Epidemiology University of North Carolina at Chapel Hill Chapel Hill North Carolina USA; ^5^ Department of Medicine Vanderbilt University Medical Center Nashville Tennessee USA; ^6^ University of Bordeaux National Institute for Health and Medical Research Research Institute for Sustainable Development Bordeaux Population Health Research Centre Bordeaux France; ^7^ TREAT Asia/amfAR The Foundation for AIDS Research Bangkok Thailand; ^8^ University of Bern Institute of Social and Preventive Medicine Bern Switzerland; ^9^ Clinical Research Education and Networking Consultancy Yaounde Cameroon; ^10^ Johns Hopkins University Bloomberg School of Public Health Baltimore Maryland USA; ^11^ Department of Pediatrics The Ryan White Center for Pediatric Infectious Disease and Global Health Indiana University School of Medicine Indianapolis Indiana USA; ^12^ Groupe Haitien d''Etude du Sarcome de Kaposi et des Infections Opportunistes (GHESKIO) Port au Prince Haiti; ^13^ Centre Medical de Suivi de Donneurs de Sang/CNTS/PRIMO‐CI Abidjan Cote D''Ivoire; ^14^ Department of Mental Health Moi Teaching and Referral Hospital Eldoret Kenya; ^15^ Centre for Infectious Disease Epidemiology & Research (CIDER) School of Public Health & Family Medicine University of Cape Town Cape Town South Africa; ^16^ Division of the AIDS Medical Information of AIDS Clinical Care National Center for Global Health and Medicine Tokyo Japan; ^17^ CTA Brazzaville Brazzaville Congo; ^18^ Department of Biomedical Informatics Vanderbilt University School of Medicine Nashville Tennessee USA; ^19^ City University of New York Institute for Implementation Science in Population Health New York New York USA; ^20^ Department of Epidemiology The Ohio State University Columbus Ohio USA

**Keywords:** mental health, HIV, integration, depression, anxiety, PTSD

## Abstract

**Introduction:**

Common mental disorders (CMDs) are highly prevalent among people with HIV. Integrating mental healthcare into HIV care may improve mental health and HIV treatment outcomes. We describe the reported availability of screening and treatment for depression, anxiety and post‐traumatic stress disorder (PTSD) at global HIV treatment centres participating in the International epidemiology Databases to Evaluate AIDS (IeDEA) Consortium in 2020 and changes in availability at sites in low‐ or middle‐income countries (LMICs) between 2016/2017 and 2020.

**Methods:**

In 2020, 238 sites contributing individual‐level data to the IeDEA Consortium and in 2016/2017 a stratified random sample of IeDEA sites in LMICs were eligible to participate in site surveys on the availability of screening and treatment for CMDs. We assessed trends over time for 68 sites across 27 LMICs that participated in both surveys.

**Results:**

Among the 238 sites eligible to participate in the 2020 site survey, 227 (95%) participated, and mental health screening and treatment data were available for 223 (98%) sites across 41 countries. A total of 95 sites across 29 LMICs completed the 2016/2017 survey. In 2020, 68% of sites were in urban settings, and 77% were in LMICs. Overall, 50%, 14% and 12% of sites reported screening with a validated instrument for depression, anxiety and PTSD, respectively. Screening plus treatment in the form of counselling was available for depression, anxiety and PTSD at 46%, 13% and 11% of sites, respectively. Screening plus treatment in the form of medication was available for depression, anxiety and PTSD at 36%, 11% and 8% of sites, respectively. Among sites that participated in both surveys, screening for depression was more commonly available in 2020 than 2016/2017 (75% vs. 59%, respectively, *p* = 0.048).

**Conclusions:**

Reported availability of screening for depression increased among this group of IeDEA sites in LMICs between 2016/2017 and 2020. However, substantial gaps persist in the availability of mental healthcare at HIV treatment sites across global settings, particularly in resource‐constrained settings. Implementation of sustainable strategies to integrate mental health services into HIV care is needed.

## 1 INTRODUCTION

Common mental disorders (CMDs), including depressive disorders, anxiety disorders and post‐traumatic stress disorder (PTSD), are highly prevalent among people with HIV (PWH) globally, including in low‐ and middle‐income countries (LMICs) [1, 2, 3, 4]. A global review of the prevalence of CMDs among PWH found that between 28% and 62% of PWH reported symptoms of a CMD, with depressive symptoms most commonly reported [1]. Other meta‐analyses have estimated the global prevalence of depression among PWH to be 31%, with a higher prevalence reported in resource‐constrained settings [5], and the global prevalence of PTSD among PWH to be 33% [6]. CMDs have been associated with poor HIV outcomes across the HIV care cascade, including delayed HIV diagnosis, suboptimal adherence to antiretroviral therapy (ART) and virologic failure [7, 8, 9, 10, 11, 12].

Evidence‐based interventions for CMD have been shown to improve the mental health of PWH [13, 14, 15]. However, mental healthcare and infrastructure remain limited worldwide, particularly across resource‐constrained settings [16, 17]. Globally, 70%–85% of people with a mental health disorder do not receive adequate mental healthcare [18, 19]. In the United States, less than half of those who need mental health services receive them [20]. Among those who receive mental healthcare, long delays between symptom onset and care receipt are common [21]. A study among PWH in South Africa estimated that the treatment gap for mental disorders was 40% among those receiving HIV care in the private sector and 96% in the public primary care sector [22]. Data on access to mental healthcare among PWH in the United States and globally remain limited [22, 23]. The World Health Organization recommends the integration of or linkage to mental health services for PWH and has developed evidence‐based guidelines for the diagnosis and management of CMDs in routine healthcare settings [24, 25]. Integrating mental healthcare into HIV care systems may serve as an effective and cost‐efficient approach to narrow the mental health treatment gap among PWH [26, 27]. Thus, it is critical to understand the extent to which CMD screening and treatment has been integrated into HIV care settings globally and how integration has changed over time.

In this paper, we (1) describe the reported availability of screening and treatment of depression, anxiety and PTSD in HIV treatment sites globally and (2) assess trends in the reported availability of screening and treatment of depression and PTSD over time at HIV sites in LMICs. A greater understanding of the integration of mental health services into HIV care can guide future research and allocation of resources to narrow the treatment gap for mental disorders among PWH.

## 2 METHODS

The International epidemiology Databases to Evaluate AIDS (IeDEA) Consortium is an international research consortium established in 2006 by the National Institutes of Health to collect global observational HIV treatment data [28, 29]. The IeDEA Consortium is comprised of HIV treatment sites across seven geographic regions: East, Central, Southern and West Africa as well as Asia‐Pacific, North America, and the Caribbean, Central and South America. To be eligible to participate in the IeDEA Consortium, HIV treatment sites must be located in a country that participates in IeDEA and have the capacity to contribute data electronically to the IeDEA Consortium.

### 2.1 2020 IeDEA site survey

The development and methods of the 2020 IeDEA site survey have been previously reported [30]. Briefly, data for the 2020 survey were collected between September 2020 and February 2021. Sites actively contributing individual‐level data to the IeDEA Consortium (*n* = 238) were eligible to participate. The 2020 survey included questions about site characteristics and the availability of screening and treatment of PWH for depression, anxiety or PTSD prior to the start of the COVID‐19 pandemic. To assess the availability of screening, sites were asked: “Are any HIV patients screened for depression?”, “Are any HIV patients screened for anxiety?” and “Are any HIV patients screened for PTSD?” For each disorder for which sites indicated that screening was available, sites were then asked to select the instrument used to screen for that disorder from a list of screening tools (Appendix). Facilities that reported that an instrument from the list provided was used for screening were categorized as *screening with a validated instrument*. Facilities that indicated screening was unavailable or was available but did not report that an instrument from the list provided was used for screening were categorized as *not screening with a validated instrument*. Screening instruments were designated “validated” if they had ever been previously validated in the peer‐reviewed literature. However, instruments may or may not have been validated in the specific country, language, population, age group or setting in which they were used. For each disorder for which facility‐based screening was reported, respondents were then asked, For patients who screen positive for depression (or anxiety or PTSD), what treatment interventions are available at this health facility?

### 2.2 2016/2017 IeDEA site survey

The methods and sampling of the 2016/2017 IeDEA site survey have been previously reported [31]. Briefly, data were collected between August 2016 and May 2017. The survey was conducted with a stratified random sample of 95 IeDEA sites located in 29 LMICs. This survey included questions on screening and treatment for depression or PTSD. The availability of anxiety screening and treatment was not assessed.

Paper and online versions of surveys were available in English and French and were piloted before the survey launch. Descriptive statistics summarize the prevalence of screening and treatment of depression, anxiety and PTSD. Chi‐squared tests were used to test differences by site characteristic in the 2020 survey. For sites that participated in both site assessments, McNemar's tests were used to compare the reported availability of screening and individual or group counselling across surveys. World Bank Income designation of the country in which the HIV clinic was located was captured as of July 2021. IeDEA site assessments have been reviewed by the Vanderbilt University Human Research Protection Program Health Sciences Committee and received a non‐human subjects determination, which means there is no human study subject so no consent or waiver of consent is needed.

## 3 RESULTS

**Figure 1:**
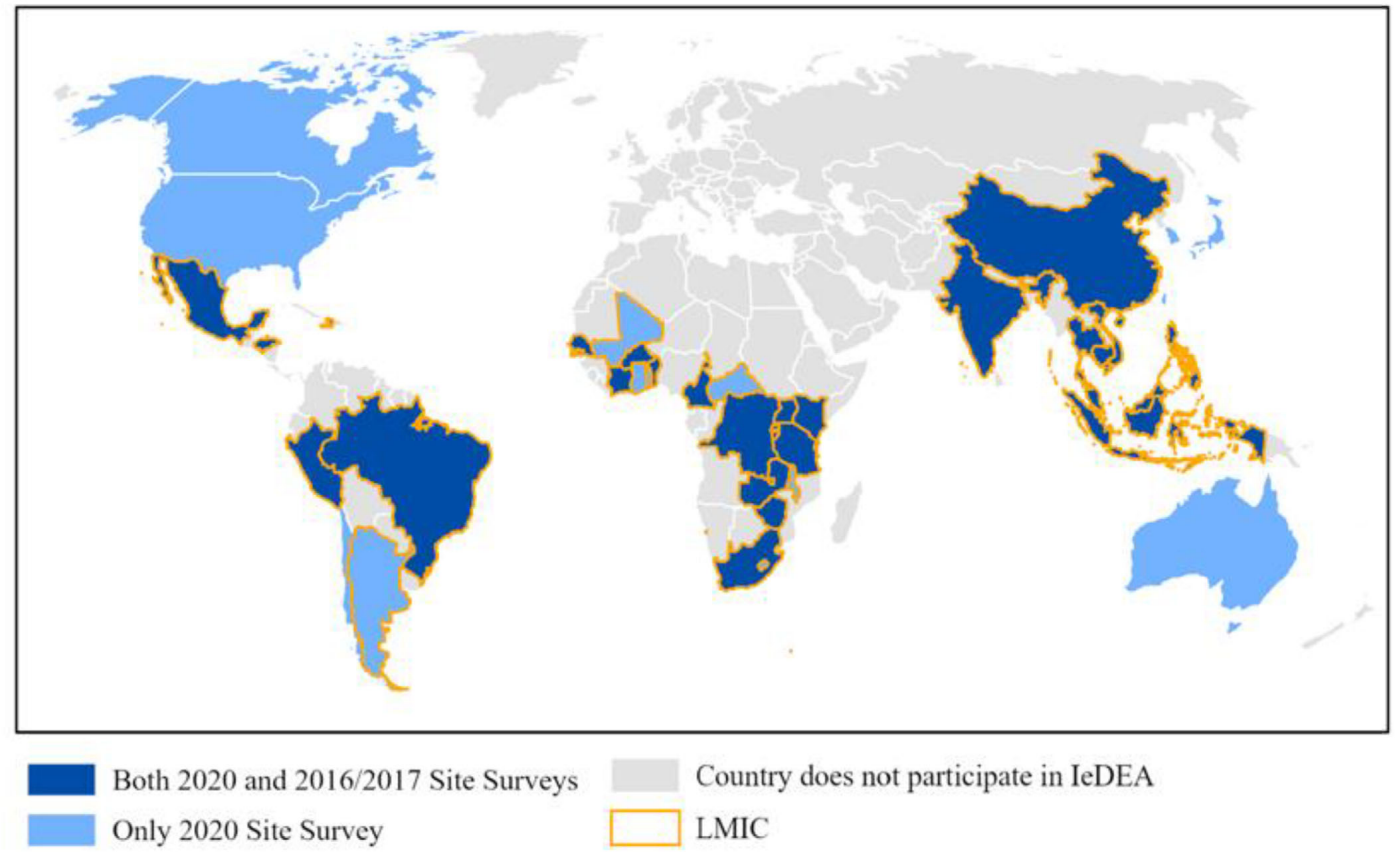
Geographic distribution of HIV treatment sites that participated in the 2016/2017 and 2020 IeDEA site surveys.

Among the 238 sites eligible to participate in the 2020 site survey, 227 (95%) participated, and mental health screening and treatment data were available for 223 (98%) sites across 41 countries. Most (85%) respondents were clinical staff or managers at the sites. Overall, 68% of sites that participated in the 2020 survey were located in urban settings, 77% were in LMICs and 38% provided HIV care to adults only (Table 1 and Figure 1).

**Table 1:** Characteristics of HIV treatment sites that completed the 2020 IeDEA site survey and both the 2020 and 2016/2017 IeDEA site surveys

	Among sites that participated in 2020 site survey *N* = 223 *n* (%)	Among sites that participated in both 2020 and 2016/2017 site surveys *n* = 68 *n* (%)
IeDEA region		
NA‐ACCORD (North America)	29 (13)	0 (0)
CCASAnet (Latin America)	8 (4)	6 (9)
Asia‐Pacific	51 (23)	24 (35)
East Africa	74 (33)	17 (25)
West Africa	14 (6)	5 (7)
Central Africa	21 (9)	10 (15)
Southern Africa	26 (12)	6 (9)
Setting		
Urban/mostly urban	150 (68)	57 (85)
Mostly rural/rural	70 (32)	10 (15)
Missing	3	1
Facility type		
Health centre	122 (56)	18 (26)
District hospital	15 (7)	6 (9)
Regional, provincial or university hospital	79 (37)	44 (65)
Missing	7	0
Population served		
Adults only	83 (38)	28 (41)
Adults and children/adolescents	111 (50)	27 (40)
Children/adolescents only	26 (12)	13 (19)
Missing	3	0
Country income group		
Low income	51 (23)	12 (18)
Lower‐middle income	84 (38)	33 (49)
Upper‐middle income	37 (17)	23 (34)
High income	51 (23)	0

### 3.1 Site characteristics and CMD screening and treatment in 2020

Approximately half (52%) of the HIV treatment sites surveyed reported screening for depression, anxiety or PTSD with a validated instrument. Screening for depression with a validated instrument was reported by 58%, 50% and 31% of sites that provided care for adults only, both adults and children/adolescents, and children/adolescents only, respectively (Table 2). Approximately one‐quarter (23%) of sites that served only adults or only children/adolescents reported screening for anxiety with a validated instrument compared to 6% of sites that served both adults and children/adolescents. Compared to sites that served only adults or both adults and children/adolescents, sites serving only children/adolescents less commonly reported screening plus medication for depression. However, sites serving both adults and children/adolescents were less likely to report screening for anxiety with a validated instrument as well as screening with a validated instrument and providing counselling or medication for anxiety. A very small proportion of sites (0%–4%) serving only paediatric clients reported screening and providing counselling or medication for PTSD. Screening plus medication for depression was more commonly reported among sites in urban compared to rural settings.

**Table 2:** Site‐level characteristics and reported availability of mental health screening and treatment at HIV treatment programmes in the IeDEA Consortium, 2020 (*n* = 223)^a^

	Depression			Anxiety			PTSD		
	Screening *n* (row %)	Screening and counselling *n* (row %)	Screening and medication *n* (row %)	Screening *n* (row %)	Screening and counselling *n* (row %)	Screening and medication *n* (row %)	Screening *n* (row %)	Screening and counselling *n* (row %)	Screening and medication *n* (row %)
Total (*N* = 233)	112 (50)	102 (46)	80 (36)	32 (14)	29 (13)	24 (11)	27 (12)	24 (11)	17 (8)
**IeDEA region**									
NA‐ACCORD (North America) (*n* = 29)	**27 (93)**	**24 (83)**	**26 (90)**	7 (24)	6 (21)	**7 (24)**	8 (28)	**8 (28)**	**7 (24)**
CCASAnet (Latin America) (*n* = 8)	**5 (63)**	**5 (63)**	**2 (25)**	1 (13)	1 (13)	**0 (0)**	0 (0)	**0 (0)**	**0 (0)**
Asia‐Pacific (*n* = 51)	**19 (37)**	**17 (33)**	**14 (27)**	12 (24)	10 (20)	**10 (20)**	3 (6)	**1 (2)**	**2 (4)**
East Africa (*n* = 74)	**39 (53)**	**35 (47)**	**20 (27)**	5 (7)	5 (7)	**3 (4)**	10 (14)	**9 (12)**	**6 (8)**
West Africa (*n* = 14)	**1 (7)**	**1 (7)**	**0 (0)**	2 (14)	2 (14)	**0 (0)**	1 (7)	**1 (7)**	**0 (0)**
Central Africa (*n* = 21)	**11 (52)**	**10 (48)**	**9 (43)**	2 (10)	2 (10)	**2 (10)**	2 (10)	**2 (10)**	**1 (5)**
Southern Africa (*n* = 26)	**10 (38)**	**10 (38)**	**9 (35)**	3 (12)	3 (12)	**2 (8)**	3 (12)	**3 (12)**	**1 (4)**
**Setting**									
Urban/mostly urban (*n* = 150)	80 (53)	74 (49)	**63 (42)**	26 (17)	23 (15)	20 (13)	20 (13)	18 (12)	13 (9)
Mostly rural/rural (*n* = 70)	32 (46)	28 (40)	**17 (24)**	6 (9)	6 (9)	4 (6)	7 (10)	6 (9)	4 (6)
Missing (*n* = 3)	0	0	**0**	0	0	0	0	0	0
**Facility type**									
Health centre (*n* = 122)	64 (52)	58 (48)	47 (39)	16 (13)	14 (11)	13 (11)	**12 (10)**	**10 (8)**	**6 (5)**
District hospital (*n* = 15)	9 (60)	8 (53)	5 (33)	2 (13)	2 (13)	1 (7)	**5 (33)**	**5 (33)**	**4 (27)**
Regional, provincial, university hospital (*n* = 79)	36 (46)	33 (42)	26 (33)	14 (18)	13 (16)	10 (13)	**9 (11)**	**8 (10)**	**6 (8)**
Missing (*n* = 7)	3	3	2	0	0	0	**1**	**1**	**1**
**Population served**									
Adults only (*n* = 83)	48 (58)	43 (52)	**43 (52)**	**19 (23)**	**16 (19)**	**15 (18)**	12 (14)	10 (12)	9 (11)
Adults and children/adolescents (*n* = 111)	55 (50)	50 (45)	**33 (30)**	**7 (6)**	**7 (6)**	**4 (4)**	14 (13)	13 (12)	8 (7)
Children/adolescents only (*n* = 26)	8 (31)	8 (31)	**4 (15)**	**6 (23)**	**6 (23)**	**5 (19)**	1 (4)	1 (4)	0 (0)
Missing (*n* = 3)	1	0	**0**	0	**0**	**0**	0	0	0

*Note*: Screening=screening with a validated instrument; counselling=individual or group counselling or therapy.

^a^Differences that were significant at *p*<0.05 level of significance are highlighted in bold.

### 3.2 Depression

Of the 223 HIV treatment sites that completed the 2020 site survey, 50% reported screening for depression with a validated instrument (Table 3). Overall, 46% reported screening and providing counselling for depression, 36% reported screening and providing pharmacological treatment, and 38% reported screening and providing peer or psychosocial support for depression. Among the 106 sites that reported screening for depression with a validated instrument and providing counselling or medication, 20% reported that patients typically had to pay fees (other than insurance co‐pays) for such treatment at their facility. Screening for depression with a validated instrument, screening plus counselling and screening plus medication were most commonly reported among sites in high‐income countries (HICs) and least commonly reported among sites located in lower‐middle‐income countries.

**Table 3:** Reported availability of CMD screening and treatment at HIV treatment sites within the IeDEA Consortium in 2020, overall and by World Bank Income designation^a^

		Work Bank income designation			
	Total *N* = 223 *n* (%)	Low *n* = 51 *n* (column %)	Lower middle *n* = 84 *n* (column %)	Upper middle *n* = 37 *n* (column %)	High *n* = 51 *n* (column %)
**Depression**					
*Any screening with validated instrument*	112 (50)	**27 (53)**	**33 (39)**	**15 (41)**	**37 (73)**
*Patient population screened with validated instrument*					
All patients with HIV	80 (36)	**18 (35)**	**25 (30)**	**9 (24)**	**28 (55)**
Specific patient populations with:					
Symptoms of mental disorders	26 (12)	5 (10)	7 (8)	5 (14)	9 (18)
Therapeutic failure	14 (6)	4 (8)	6 (7)	2 (5)	2 (4)
Suboptimal ART adherence	20 (9)	7 (14)	7 (8)	3 (8)	3 (6)
Other	3 (1)	2 (4)	0 (0)	1 (3)	0 (0)
*Screening with validated instrument and treatments available*					
Counselling	102 (46)	**24 (47)**	**30 (36)**	**14 (38)**	**34 (67)**
Medication	80 (36)	**18 (35)**	**18 (21)**	**9 (24)**	**35 (69)**
Peer or psychosocial support	85 (38)	22 (43)	28 (33)	10 (27)	25 (49)
None	3 (1)	1 (2)	1 (1)	0 (0)	1 (2)
**Anxiety**					
*Any screening with validated instrument*	32 (14)	**2 (4)**	**9 (11)**	**8 (22)**	**13 (25)**
*Patient population screened with validated instrument*					
All patients with HIV	18 (8)	**0 (0)**	**6 (7)**	**5 (14)**	**7 (14)**
Specific patient populations with:					
Symptoms of mental disorders	13 (6)	2 (4)	3 (4)	2 (5)	6 (12)
Therapeutic failure	5 (2)	2 (4)	1 (1)	0 (0)	2 (4)
Suboptimal ART adherence	5 (2)	2 (4)	1 (1)	0 (0)	2 (4)
Other	3 (1)	1 (2)	0 (0)	1 (3)	1 (2)
*Screening with validated instrument and treatments available*					
Counselling	29 (13)	**2 (4)**	**8 (10)**	**8 (22)**	**11 (22)**
Medication	24 (11)	**2 (4)**	**3 (4)**	**7 (19)**	**12 (24)**
Peer or psychosocial support	27 (12)	**2 (4)**	**8 (10)**	**5 (14)**	**12 (24)**
None	0 (0)	0 (0)	0 (0)	0 (0)	0 (0)
**PTSD**					
*Any screening with validated instrument*	27 (12)	**3 (6)**	**13 (15)**	**1 (3)**	**10 (20)**
*Patient population screened with validated instrument*					
All patients with HIV	13 (6)	1 (2)	7 (8)	0 (0)	5 (10)
Specific patient populations with:					
Symptoms of mental disorders	12 (5)	1 (2)	6 (7)	0 (0)	5 (10)
Therapeutic failure	6 (3)	0 (0)	4 (5)	0 (0)	2 (4)
Suboptimal ART adherence	7 (3)	1 (2)	4 (5)	0 (0)	2 (4)
Other	2 (1)	1 (2)	0 (0)	1 (3)	0 (0)
*Screening with validated instrument and treatments available*					
Counselling	24 (11)	3 (6)	11 (13)	1 (3)	9 (18)
Medication	17 (8)	**2 (4)**	**6 (7)**	**0 (0)**	**9 (18)**
Peer or psychosocial support	21 (9)	3 (6)	10 (12)	0 (0)	8 (16)
None	0 (0)	(0)	(0)	(0)	(0)

^a^ Differences that were significant at *p*<0.05 level of significance are highlighted in bold.

### 3.3 Anxiety

Overall, 14% of sites surveyed in 2020 reported screening for anxiety using a validated instrument with only 8% reporting screening all patients with HIV for anxiety with a validated instrument. In 2020, 13% of sites surveyed reported both screening and counselling for anxiety, 11% reported screening and psychiatric medication for anxiety, and 12% reported screening plus peer or psychosocial support for anxiety. Among the 31 sites that reported screening and providing counselling or medication for anxiety, 35% reported that clinic patients typically had to pay fees (other than insurance co‐pays) for such treatment at their facility. Screening for anxiety with a validated instrument was most commonly reported by sites in HICs and least commonly reported among sites in low‐income countries.

### 3.4 PTSD

Overall, 12% of sites reported screening for PTSD using a validated instrument with 6% reporting screening all patients with HIV for PTSD with a validated instrument. In 2020, 11% of sites reported both screening for PTSD and providing counselling, 8% reported screening for PTSD and providing medication, and 9% reported screening for PTSD and providing peer or psychosocial support. Among the 25 sites that reported screening for PTSD and providing counselling or medication, 16% reported that clinic patients typically had to pay fees (other than insurance co‐pays) for such treatment at their facility. Sites in HICs more commonly reported screening for PTSD with a validated instrument and screening plus medication compared to sites in LMICs.

### 3.5 Availability of psychiatric medications

In the 2020 survey, nearly all sites (94%; *n* = 209) reported that there was a pharmacy at their facility (Table 4). Among sites that reported an on‐site pharmacy, approximately two‐thirds reported that mood stabilizers, antipsychotic medications and tricyclic antidepressants were available (66%, 65% and 62%, respectively). However, 19%–24% of these sites also reported supply disruptions of these medications. Half of sites with an on‐site pharmacy reported that benzodiazepines were available. Selective serotonin reuptake inhibitors and serotonin and norepinephrine reuptake inhibitors were available at 36% and 32% of sites, respectively.

**Table 4:** Reported availability of psychiatric medications among HIV treatment sites with an on‐site pharmacy in 2020, IeDEA Consortium (*n* = 209)

Medication available at on‐site pharmacy	Yes *n* (%)
Serotonin and norepinephrine reuptake inhibitors (SNRIs)	67 (32)
Selective serotonin reuptake inhibitors (SSRIs)	75 (36)
Benzodiazepines	105 (50)
Tricyclic antidepressants	129 (62)
Antipsychotic medications	135 (65)
Mood stabilizers	137 (66)
**Reported stockout (among those that reported each medication)**	
SNRI (*n* = 67)	3 (4)
SSRI (*n* = 75)	5 (7)
Benzodiazepines (*n* = 105)	22 (21)
Tricyclic antidepressants (*n* = 129)	24 (19)
Antipsychotic medications (*n* = 135)	32 (24)
Mood stabilizers (*n* = 137)	27 (20)

### 3.6 Changes in reported availability of mental health services at HIV sites in LMICs between 2016/2017 and 2020

Only 68 of the 95 LMIC sites (from 27 countries) participated in both the 2016/17 and 2020 site surveys. Overall, 85% of sites that participated in both surveys were located in urban settings, and 41% provided HIV care to adults only. It is important to note that the 2016/2017 survey only asked about “any screening” for depression and PTSD, rather than screening with a validated instrument. For depression, any screening (i.e. not limited to screening with a validated instrument) was more commonly reported in 2020 compared to 2016/2017 (Table 5). However, the reported availability of screening and group counselling for depression was significantly lower in 2020 compared to 2016/2017. No statistically significant differences in the reported availability of screening or treatment for PTSD were observed between 2016/2017 and 2020.

**Table 5:** Reported availability of screening and treatment for depression and PTSD among HIV treatment sites in LMICs that participated in the 2016/2017 and 2020 surveys, IeDEA Consortium (*n* = 68)

	2016/2017 site survey (*N* = 68) *n* (column %)	2020 site survey (*N* = 68) *n* (column %)	McNemar's test (*p*‐value)
Depression			
Screening	40 (59)	51 (75)	3.90 (0.048)
Screening and individual counselling	33 (49)	41 (60)	2.29 (0.131)
Screening and group counselling	24 (35)	14 (21)	4.17 (0.041)
Screening and both individual and group counselling	24 (35)	13 (19)	5.26 (0.022)
PTSD			
Screening	12 (18)	20 (29)	2.67 (0.152)
Screening and individual counselling	10 (15)	16 (24)	1.64 (0.286)
Screening and group counselling	8 (12)	6 (9)	0.33 (0.774)
Screening and both individual and group counselling	8 (12)	6 (9)	0.33 (0.774)

*Note*: Screening = “Any Screening,” (e.g. not limited to screening with a validated instrument). For comparisons with cell sizes <5, exact methods were used to calculate *p*‐values.

**Figure 2:**
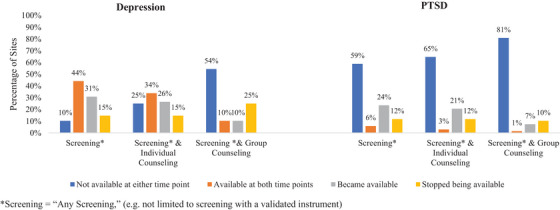
Changes in the reported availability of depression and PTSD screening and treatment at HIV treatment sites participating in the 2016/2017 and 2020 IeDEA site surveys (*N* = 68).

Overall, 10% of facilities that participated in both surveys did not report depression screening at either time point, 44% reported availability of depression screening at both time points, 31% reported that depression screening became available (screening available in 2020, but not 2016/2017) and 15% reported screening stopped being available (screening available in 2016/2017, but not 2020) (Figure 2). When examining the combined availability of screening and individual counselling for depression, 25% reported that such services were unavailable at both time points. When assessing the combined availability of screening and group counselling for depression, 54% reported that such services were unavailable at both time points and 10% reported that such services were available at both time points.

Over half (59%) of facilities participating in both surveys reported that PTSD screening was unavailable at both time points. When examining the combined availability of screening and individual counselling for PTSD, 65% reported that such services were unavailable at both time points and just 3% reported that such services were available at both time points. Similarly, most (81%) facilities reported that screening and group counselling for PTSD was unavailable at both time points, and just 1% reported that such services were available at both time points.

## 4 DISCUSSION

We surveyed 223 IeDEA sites in 2020 to assess the reported availability of screening and treatment for depression, anxiety and PTSD for PWH receiving HIV care at these facilities. Significant gaps persist in the availability of screening and treatment of CMDs across global regions, particularly for anxiety and PTSD. Disparities in CMD screening persist between sites serving adult and paediatric populations, urban and rural settings, and sites located in HICs and LMICs. Reported availability of screening for depression increased, while the availability of group counselling for depression decreased from 2016/2017 to 2020.

The current analysis highlights that the integration of mental healthcare into HIV care remains limited across global settings. Less than half of IeDEA sites surveyed in 2020 reported screening with a validated instrument and providing some form of treatment for depression, anxiety or PTSD. Disparities between HICs and LMICs were evident. Similar disparities were noted in the full 2016/2017 survey [31]. The mental health treatment gap globally and in resource‐constrained settings has been previously documented [23, 27]. Many barriers to integrating evidence‐based mental healthcare into HIV care in resource‐constrained settings have been identified, including the high prevalence of HIV in some settings, over‐burdened healthcare systems, limited psychiatric human resources, infrastructure, funding or supportive policies, and HIV‐ and mental illness‐related stigma [23, 32]. Promising strategies to address these challenges and provide integrated mental health and HIV care have been developed, including task‐shifting and stepped‐care models, transdiagnostic mental health treatments and the use of technology, to increase access to protocolized evidenced‐based care [23, 33]. In recent years, motivated in part by the COVID‐19 pandemic, the availability and reach of telehealth mental health services, including app‐based mental healthcare, has increased. Particularly in areas with few trained mental health providers, the feasibility, acceptability and sustainability of telehealth mental health services should be explored. Implementation science research is needed to identify feasible and sustainable strategies to integrate mental health services into HIV care across the globe, but particularly in LMIC settings where mental healthcare is most scarce [34, 35].

Among sites in LMICs that participated in both surveys, 31% of sites reportedly began screening for depression and 24% reportedly began screening for PTSD between the 2016/2017 and 2020 surveys. Increased integration of screening in these sites may represent increasing awareness among HIV service providers or healthcare or government officials of the burden and impact of untreated mental disorders among PWH. However, a number of sites also reportedly stopped screening or providing treatment for depression and PTSD between 2016/2017 and 2020. In resource‐constrained settings where mental healthcare expansion may be donor‐ or research‐driven, challenges sustaining integrated services have been documented [36]. Longer‐term research into the sustainability of mental health treatment after evaluation or external funding has ended remains scarce. Research to understand factors that influence the expansion of mental healthcare for PWH and identify feasible and effective strategies for implementing and sustaining mental healthcare in resource‐constrained and rural settings is urgently needed. Research that investigates the long‐term sustainability of mental health service integration is especially needed, particularly among sites that discontinue screening programmes or specific treatment options. The sustainable integration of mental healthcare into HIV care presents ongoing challenges across global settings.

Children and adolescents, including children and adolescents with HIV, face a severe mental health treatment gap [37]. A review of CMDs in youth with HIV in sub‐Saharan Africa estimated a prevalence of major depression between 16% and 41% and a prevalence of anxiety disorder of 46%[38]. Our analysis found that the reported availability of screening and treatment for depression was substantially lower among sites serving only children/adolescents compared to sites serving adults only or both adult and paediatric clients, highlighting a pronounced mental health treatment gap at paediatric HIV care settings. Children and adolescents with HIV face unique mental health stressors and challenges and require evidence‐based mental health screening and paediatric‐appropriate treatment approaches [37]. While some evidence‐based mental health programmes have been developed for paediatric populations, including school and community‐based programming and cognitive behavioural therapy [39, 40, 41], the paediatric mental healthcare evidence base is lagging, particularly for children and adolescents with HIV and in resource‐constrained settings [37]. Hybrid effectiveness‐implementation research into paediatric mental healthcare programming is urgently needed to hasten the process of delivering evidence‐based mental healthcare to this particularly vulnerable population.

Across the globe, and particularly in resource‐constrained settings, barriers and facilitators persist to the provision of pharmacological and psychological treatment for CMDs. Evidenced‐based counselling requires training, supervision and substantial time for patient and provider. In addition, fees for mental health treatment were commonly reported in many IeDEA sites, creating an additional barrier which may hinder treatment initiation and retention. The provision of pharmacologic treatment is commonly hindered by supply chain challenges, limited mental healthcare budgets, limited availability of first‐line medications, and required clinical acumen and supervision [36, 42, 43]. Many challenges associated with the provision of psychiatric medication may be less salient in HICs, where HIV care sites often have access to a range of pharmacological treatments, including those with more favourable side effect profiles. Evidence‐based interventions to integrate the provision of pharmacological treatment for CMDs into HIV care in resource‐constrained settings have been developed, notably measurement‐based care models that can be implemented with non‐psychiatric specialists [44, 45, 46, 47, 48]. Effectively tailoring mental health interventions requires understanding providers’ and patients’ needs and preferences. Research to identify patients' and providers’ needs and preferences, through conjoint analysis or discrete choice experiments, may provide useful insight [49]. Further, efforts to incorporate mental health services into HIV care must consider that the most acceptable and effective mental healthcare programmes may include multiple treatment options to accommodate a range of patients’ preferences.

There are inherent limitations to this study. Of note, 223 sites participated in the 2020 survey and 68 sites participated in both surveys. As a result, a number of comparisons were made among a relatively small sample. Screening and treatment practices were self‐reported and not independently verified. Thus, responses may reflect social desirability, an over‐reporting of services or limited knowledge of existing mental health services. Further, for sites that reported providing medication as treatment for depression, anxiety or PTSD, we lack detail on the availability or accessibility of this medication in terms of where or how consistently it could be acquired as well as its cost. Greater information about the consistent availability and accessibility of such medication is needed. HIV treatment sites that participate in IeDEA may be relatively well‐resourced which has implications for generalizability. Changes in questions between surveys sometimes hindered our ability to make direct comparisons, particularly in relation to the availability of screening for depression or PTSD with a validated instrument. While the screening instruments referenced in this study are widely used and have undergone formal validation processes in multiple contexts, they may or may not have been validated among PWH or across all of the specific countries, age groups, languages or settings where they were employed. Further, there is likely variability in how screening and treatment are delivered across settings, as well as the degree to which these services are grounded in evidence‐based practice.

## 5 CONCLUSIONS

While screening and treatment of CMDs is increasingly integrated into HIV services, mental health services at HIV treatment sites remain limited and disparities between HIC and LMIC settings persist. There is a clear need to expand access to mental healthcare for PWH and to identify strategies to integrate evidence‐based screening and treatment for CMDs into HIV care.

## COMPETING INTERESTS

The authors declare no competing interests.

## AUTHORS’ CONTRIBUTIONS

AMP, KL, CWW, SND and DN designed the survey. CWW, SND and DN coordinated data collection. MS and MR performed the data analysis and interpretation. AMP and MS drafted the manuscript. AMP, MS, MR, CWW, CB, JR, ADH, RA, KNA, LE, WP, AM, EK, MT, JT, DN, AF, SND, DN and KL revised the manuscript. All authors read and approved the final manuscript.

## FUNDING

The International Epidemiology Databases to Evaluate AIDS (IeDEA) is supported by the U.S. National Institutes of Health's National Institute of Allergy and Infectious Diseases, the *Eunice Kennedy Shriver* National Institute of Child Health and Human Development, the National Cancer Institute, the National Institute of Mental Health, the National Institute on Drug Abuse, the National Heart, Lung, and Blood Institute, the National Institute on Alcohol Abuse and Alcoholism, the National Institute of Diabetes and Digestive and Kidney Diseases, and the Fogarty International Center : Asia‐Pacific, U01AI069907; CCASAnet, U01AI069923; Central Africa, U01AI096299; East Africa, U01AI069911; NA‐ACCORD, U01AI069918; Southern Africa, U01AI069924; West Africa, U01AI069919. Informatics resources are supported by the Harmonist project, R24AI24872. This research was also supported in part by NIMH grant K01MH114721 and NICHD grant P2CHD050924.

## DISCLAIMER

The content of this publication is solely the responsibility of the authors and does not necessarily represent the official views of any of the governments or institutions mentioned above.

## Data Availability

Complete data for this study cannot be posted in a supplemental file or a public repository because of legal and ethical restrictions. The Principles of Collaboration under which this multi‐national consortium was founded and the regulatory requirements of the different countries’ IRBs require the submission and approval of a project concept sheet. The data held by the IeDEA Consortium are available to other investigators, but must be based on a concept note describing the planned analysis and approved by the regional Steering Groups and, if analyses involve several regions, by the IeDEA Executive Committee (Chairperson; Annette Sohn, MD; email:annette.sohn@treatasia.org).

## Instruments included in 2020 IeDEA survey, by disorder

**Table:**

Disorder	Instruments
Depression	Beck Depression Inventory, Center for Epidemiologic Studies Depression Scale (CES‐D), Hamilton Rating Scale for Depression (HAM‐D), Hospital Anxiety and Depression Scale (HAD), Patient Health Quesitonnaire‐2 (PHQ‐2), Patient Health Questionnaire‐9 (PHQ‐9)
Anxiety	Beck Anxiety Inventory (BAI); General Anxiety Disorder 7 (GAD‐7); Hospital Anxiety and Depression Scale (HAD); State‐Trait Anxiety Inventory (STAI)
PTSD	Life Event Checklist; Primary Care PTSD Screening (PC‐PTSD); PTSD Checklist – Civilian version (PCL‐C); PTSD Checklist for DSM‐5 (PCL‐5); Short PTSD Rating Interview (SPRINT); Trauma Screening Questionnaire (TSQ)
